# “I'm not a freshi”: Culture shock, puberty and growing up as British-Bangladeshi girls

**DOI:** 10.1016/j.socscimed.2020.113058

**Published:** 2020-08

**Authors:** Lauren C. Houghton, Rebecca Troisi, Marni Sommer, Hormuzd A. Katki, Mark Booth, Osul A. Choudhury, Kate R. Hampshire

**Affiliations:** a Department of Epidemiology, Mailman School of Public Health, 722 W 168th St, New York, NY, 10032, USA; b Faculty of Medical Sciences, Newcastle University, Framlington Place, Newcastle Upon Tyne, NE2 4HH, United Kingdom; c Department of Sociomedical Sciences, Mailman School of Public Health, 722 W 168th St, New York, NY, 10032, USA; d Division of Cancer Epidemiology and Genetics, National Cancer Institute, 9609 Medical Center Dr, Rockville, MD, 20850, USA; e Sylhet MAG Osmani Medical College, Sylhet, Bangladesh; f Department of Anthropology, Durham University, South Road, Durham, DH1 3LE, United Kingdom

**Keywords:** Puberty, Migration, Mixed-methods, Androgens, Biocultural

## Abstract

Early puberty is a risk factor for adult diseases and biomedical and psychosocial research implicate growth (in height and weight) and stress as modifiable drivers of early puberty. Seldom have studies examined these drivers simultaneously or concurrently using quantitative and qualitative methods. Within the context of migration, we used mixed-methods to compare growth, stress and puberty in a study of 488 girls, aged 5–16, who were either Bangladeshi, first-generation migrant to the UK, second-generation migrant, or white British (conducted between 2009 and 2011). Using a biocultural framework, we asked the questions: 1) Does migration accelerate pubertal processes? 2) What biocultural markers are associated with migration? 3) What biocultural markers are associated with puberty? Girls self-reported pubertal stage, recalled 24-h dietary intake, and answered questions relating to dress, food, and ethnic identity. We collected anthropometrics and assayed saliva specimens for dehydroepiandrosterone-sulfate (DHEA-S) to assess adrenarcheal status. Our findings demonstrate that first-generation migrants had earlier puberty than second-generation migrants and Bangladeshi girls. British style of dress did not increase with migration, while dietary choices did, which were reflected in increasing body mass index. However, the widely-used phrase, “I'm proud of my religion, but not my culture” demonstrated that ethnic identity was aligned more with Islamic religion than ‘Bangladeshi culture.’ This was epitomized by wearing the hijab, but denial of eating rice. The social correlates of puberty, such as ‘practicing’ wearing the hijab and becoming ‘dedicated to the scarf,’ occurred at the same ages as adrenarche and menarche, respectively, among first-generation girls. We suggest that the rejection of ‘Bangladeshi culture’ might be a source of psychosocial stress for first-generation girls, and this may explain elevated DHEA-S levels and early puberty compared to their second-generation counterparts. Our results support a biocultural model of adolescence, a period for biological embedding of culture, when biological and psychosocial factors adjust developmental timing with potential positive and negative implications for long-term health.

## Highlights

• Compares puberty in migrant Bangladeshi girls in the UK using mixed-methods.
• Argues that stigma may explain early maturation in first-generation migrants.
• Reveals overlapping ages of biological and cultural puberty in migrant girls.
• Generates new biocultural hypotheses via hormonal, growth and social stigma data.

## 1 Introduction

Puberty marks a transition to maturity encompassing unparalleled biosocial changes that are only second in rate of growth (in height and weight) to those experienced during infancy. The timing and qualitative features of the pubertal transition appear to have long-lasting effects; for instance, early biological development of puberty is linked to the risk of later adult diseases, such as cardiovascular disease and breast cancer (Bodicoat et al., 2014; Worthman, 1999). In relation to social development, adolescence is a sensitive period for specific forms of sociocultural learning and the acquisition of skill sets that are needed to transition successfully towards adult roles (Blakemore and Mills, 2014). Concerns about the later health risks of individuals with precocious puberty (Herman-Giddens et al., 1997), populations with higher prevalence of early puberty, and secular declines in the average age of puberty (Kaplowitz, 2006), have led to investigations of what drives this early development (Herman-Giddens et al., 2004). Established drivers of pubertal timing include ethnicity; growth in height, weight, and BMI; and stress. (Greenspan and Deardorff, 2014 ) Variation in age at pubertal onset within ethnic groups by migration status challenges the notion that genetic factors explain differences in pubertal timing; rather, the quality of early environments characterized by differential growth in height, weight and BMI and psychosocial stress consistently emerge as major factors driving pubertal timing (Perkins et al., 2016). Part of the trend toward earlier puberty may be due to increasing obesity (Lee and Styne, 2013; Biro et al., 2013). However, population declines in age at pubertal onset began before the obesity epidemic, suggesting that other drivers are also at play (McDonald et al., 2016). Various forms of psychosocial stress, such as maternal depression and father absence, have also been associated with earlier pubertal timing (Belsky et al., 2015; Deardorff et al., 2011). In relation to social aspects of puberty, in 2018 Carol Worthman elegantly illustrated how between the mid 1800s to the early 2000s, the age at biological maturation declined while the age at social maturation (measured as years in school) increased, arguing that adolescence is becoming a longer transition within the lifecourse (Worthman and Trang, 2018). This mismatch between the ages of biological and social maturation is reinforced by the different approaches disciplines take when studying puberty. Despite the biosocial nature of puberty, few studies have taken a biocultural approach when comparing the timing, drivers, and experience of puberty across populations.

Studying biological and social aspects of puberty within the context of migration allows for a biocultural investigation of embodiment: how people “literally embody, biologically, the multilevel dynamic and constituted societal and ecologic context within which [they] live …” (Krieger, 2016). Theoretically, both puberty and migration reflect processes of embodiment. Older age at puberty is associated with social deprivation through pathways of embodiment including malnutrition and childhood infections arising from crowding and inadequate sanitation (Krieger and Davey Smith, 2004). Migration intersects with embodiment in that it can affect health through direct or indirect effects on biology (Fox et al., 2017). For example, migration may present direct exposure to a specific carcinogen, or migration accompanied by financial insecurity in the host country could indirectly affect health through lack of access to care. Investigating puberty (a biocultural transition for an individual) within the context of migration (a biocultural transition at the population level) allows for identification of biological and social drivers of pubertal timing, and how this period of plasticity might be leveraged beneficially for future health.

Puberty is not a single event but rather a series of changes including, often in this order for girls: adrenarche, thelarche, and menarche (Houghton et al., 2014). Adrenarche is marked by increased levels of dehydroepiandrosterone-sulfate (DHEAS) (Reiter et al., 1977), the most abundant androgen in circulation, and entails the maturation of the hypothalamic-pituitary-adrenal (HPA) axis, also known as the stress axis. Occurring between the ages of 6 and 8 years, on average in both boys and girls, adrenarche corresponds with advances in brain development, learning and socialization, and cross-culturally begins the juvenile period when children are recognized as able to handle more social responsibilities, such as sibling care, attending school, etc. (Campbell, 2006a) Thelarche is the onset of breast development and typically occurs between the ages of 8–10 in girls (Biro et al., 2003). Menarche, when a girl has her first menstrual period, is the easiest stage to document and therefore the most widely used marker of pubertal maturation, but is usually one of the last changes in puberty, occurring up to 4 years after thelarche. Because puberty is a series of physical, hormonal, and neurological changes that occur over time, it is a period of plasticity when environmental cues can shape subsequent social and biological trajectories.

**Fig. 1:**
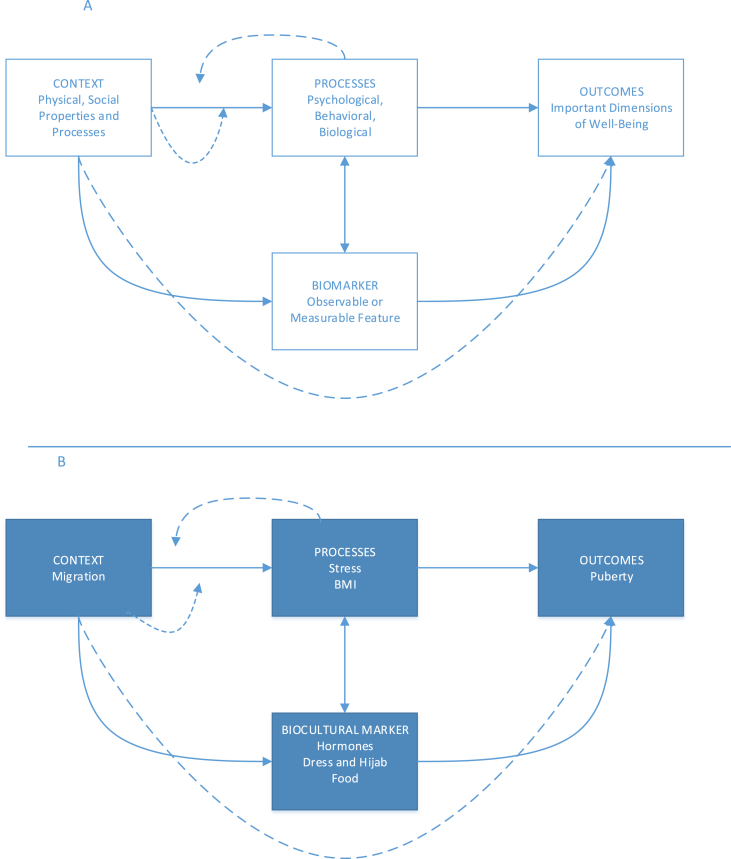
Generalized Model for Bio(cultural)markers, adapted from Worthman and Costello (2009). Panel A is Worthman and Costello's biomarker model and Panel B is our adapted biocultural marker model for puberty.

In this study, we modified Carol Worthman's biocultural framework (Worthman and Costello, 2009) (Fig. 1A) to explore the timing, drivers, and experiences of puberty. To explain differences in pubertal timing within the context of migration, we integrate qualitative and quantitative methods and identify biocultural markers associated with migration, puberty, and the mediating processes of stress and growth. (Fig. 1B). We address the following research questions: 1) Does migration accelerate pubertal processes?? 2) What biocultural markers are associated with migration? 3) What biocultural markers are associated with puberty, specifically, how do ages of biological and cultural of markers puberty compare across the different migrant groups?

## 2 Methods

### 2.1 Study design

#### 2.1.1 Study populations

In this cross-sectional study, the ABBY (Adolescence among Bangladeshi and British Youth) Project, we collected both biological and cultural data surrounding the experience of puberty among Bangladeshi, British-Bangladeshi and white British girls aged 5–16 years across two field sites: London, UK, and Sylhet, Bangladesh, between the years 2009–2011. We categorized girls into four groups defined by environment and ethnicity: 162 Bangladeshi (B; born and living in Bangladesh), 40 first-generation migrants (BB1; born in Bangladesh but migrated to the UK), 159 second-generation migrants (BB2; born and living in the UK, whose parents migrated from Bangladesh), and 48 white British (WB; born and living in the UK of European ethnicity), in this order collectively referred to as the ‘migration scale’. (Houghton et al., 2014).

#### 2.1.2 Recruitment

Girls were recruited using convenience sampling (Bryman, 2008) from eight primary and two secondary schools in East London with a high percentage of Bangladeshi pupils; at least 50% and up to 90% of the students in each school came from British-Bangladeshi households. Initially, schools were contacted via drop-in visits and through distributing written project materials. From this initial recruitment effort, two schools (UK Primary School 1 and UK Secondary School 1) agreed to participate. UK Secondary 1 belonged to a partnership of schools that consisted of several primary schools designated as feeder schools; four of these primary schools (UK Primary School 2, 3, 4 and 5) agreed to take part in the study. Additional schools were recruited through key contacts. All heads of the schools granted permission for their pupils to participate in the ABBY Project. Initial contact with participants occurred during school time, in women-only settings, where study staff explained the ABBY Project to all girls in Years 1–10, using photographic information sheets, samples of collection materials and an icebreaker activity. Staff gave girls information sheets and parental consent forms. Study staff later visited the schools to collect the signed consent forms. Before the interviews began, the interviewer explained the project again and sought assent from the child. In Sylhet, we recruited from seven private and semi-private/semi-government schools because of their high concentration of middle to lower-middle class families. These families were more likely to have relatives who had migrated to the UK, and were thus presumed to be more comparable on confounding factors with first- and second-generation girls recruited in the UK.

The ABBY Project received ethics approval from the the Department of Anthropology Durham University Ethics Committee and the Sylhet MAG Osmani Medical College. The Office of Human Research Subjects at the National Cancer Institute issued an Institutional Review Board (IRB) exemption based on the other approvals. Tower Hamlets Local Authority also reviewed and approved the study. We obtained informed consent from parents and assent from girls using picture-based information sheets.

## 3 Overview of mixed-methods approaches used

We took an integrated mixed-methods approach (Zhang and Creswell, 2013), assessing most variables using both quantitative and qualitative methods. We collected quantitative data from individual participants through interviewer-administered, structured questionnaires and biospecimens. In the UK, we collected qualitative data through participant observation and focus group discussions with girls aged 14–15 years (n = 20) over a period of 36 weeks during an after-school club in one secondary school. LCH conducted all participant observation over an 18-month period. In Sylhet, study staff held one-time focus groups with different age groups and discussed findings after each focus group with LCH, who then took notes to compare with UK-based focus groups. Below we describe the quantitative and qualitative methods we used to assess each construct in the study and their integration.

### 3.1 Quantitative methods

#### 3.1.1 Puberty

We collected saliva samples at the time of interview for measurement of DHEA-S levels as described previously (Houghton et al., 2014). We considered girls to have reached adrenarche when salivary DHEA-S levels were >400 pg/ml (Havelock et al., 2004). We assessed thelarche by self-report using a modified version of the pubertal development scale (PDS); the PDS equivalent to Tanner Stage 2 corresponds with breast budding (Petersen et al., 1988; Marshall and Tanner, 1969). The girls reported whether they had reached menarche at the time of interview and if so, the age (years and month) when it occurred.

#### 3.1.2 Biocultural markers

We selected biocultural markers based on prior studies that used language, friendship and dress as measures to explore acculturation and identity among youth (Bhui et al., 2005). Also, food has been suggested as a marker of British-South Asian acculturation (Gilbert and Khokhar, 2008). We assessed biocultural markers using a structured questionnaire asking about self-reported ethnicity (British, Bangladeshi, or British-Bangladeshi), language spoken at home (English or Bangla), and preferences in friends in relation to ethnicity (*Do you have many good friends who belong to your ethnic group? Do you have any good friends who belong to other ethnic groups? Response options: 1) None, 2) Some, 3) Quite a lot, 4) Most of them*) and choice of dress (*Is your choice in clothes similar to people from your ethnic group? Is your choice in clothes similar to people from other ethnic groups? Response options: 1) No, 2) A little like them, 3) Quite a lot like them, 4) Mostly like them*). We collapsed the response options into binary variables (1 vs 2+).

##### 3.1.2.1 Food

To assess the type of foods eaten by the girls, we designed a 24-h food recall to capture consumption at seven specific meals and meal times including: breakfast, a mid-morning break during school time, lunch, snacks on the way home from school, at home after school, the evening meal and food eaten before bedtime. The latter specifically captured the late night meal typically eaten by Bangladeshi families (Lofink, 2012). The dietary recall also included supplementary questions regarding typical food preferences.

##### 3.1.2.2 Growth

Anthropometric measurements, including height and weight were taken while the participant was clothed, but without shoes and bulky outer clothing. We calculated the body mass index (BMI) by dividing weight in kilograms by stature in meters squared. BMI z-scores were calculated according to the method described by Cole et al. (Cole and Green, 1992) and compared to UK 1990 growth references (Cole et al., 1998).

### 3.2 Qualitative methods

#### 3.2.1 Puberty

We had no preconceived social correlates of puberty. Participant observations and focus groups revealed that dress and hijab, measures we originally included as biocultural markers of migration, were also salient social correlates of puberty. There may have been other social correlates of puberty that we did not identify because they did not coincide with predetermined markers of migration.

#### 3.2.2 Biocultural markers

##### 3.2.2.1 Dress (clothes and hijab)

During 18 months of fieldwork in east London and Sylhet, we kept a field journal to record observations. We conducted participant observations primarily through facilitating weekly after-school clubs in the UK. The after-school clubs provided opportunities to explore the experience of socially maturing through informal discussions and focus groups. Girls self-selected to join the after-school club and all of those that participated also completed a structured questionnaire. In Bangladesh, time constraints and language barriers made it difficult to facilitate after-school clubs. Alternatively, we organized focus groups in Sylhet either as part of supplementary science classes or during existing school activities. We selected girls to participate in the focus groups based on age after they completed the structured questionnaire.

In the UK, to explore the significance of wearing hijab, we held two focus group discussions (total n = 12). Questions included: “When should a girl wear a scarf?” “Why do you wear or not wear a scarf?” In Bangladesh, Sylheti-speaking research assistants facilitated two focus groups (total n = 12) concerning choice of clothing and how this changed as they become older. Study staff made notes during the focus groups and recorded full field notes after the sessions (Lofland and Lofland, 2006).

##### 3.2.2.2 Food

In-depth perceptions and attitudes towards food, including Bengali versus English cuisine and associated consumption patterns, were explored through two focus groups in the UK during the afterschool clubs (total n = 12).

## 4 Analytical procedures

### 4.1 Quantitative data analysis

We compared the timing of adrenarche, thelarche, and menarche among Bangladeshi, first and second generation migrants and white British girls using flexible Weibull regression models for current status data survival analysis using STATA Version 11.2 (STATA Corporation, College Station, Texas, USA). This method uses age as the timescale and can account for interval-, left- and right-censored data, which Cox Proportional hazards methods cannot accommodate (Royston, 2001). Weibull models produce both predicted group medians as well as hazard ratios (HR) with 95% Confidence Intervals (CI), with HRs greater than 1 indicating earlier puberty. For each pubertal outcome, we ran three Weibull models (Model 1:test for trend using the continuous migration scale variable as the predictor, Model 2: pairwise comparisons between Bangladeshi and the other groups using migration scale as categorical predictors, and Model 3: pairwise comparisons between the first generation and second generation girls using a dummy variable as the predictor). From Model 2, we predicted the median age of puberty for each group. When interpreting our results, we considered statistical significance as well as overall patterns in the estimated medians. Also using Weibull models, we estimated the association between BMI z-scores and the timing of adrenarche, thelarche, and menarche in all girls, and in models stratified by migrant group to test if the association between BMI and pubertal timing differed among the groups. Positive beta coefficients represent earlier pubertal timing per unit increase in BMI z-score. This stratified analysis allowed us to differentiate the effect of stress from the effect of growth on pubertal timing since we argue that first generation girls are a uniquely stressed group.

We compared the biocultural markers (language, dress, friends, food) across the four migrant groups using chi-squared tests. We analyzed the dietary data by creating a binary variable as to whether the girls reported eating rice during the 24-hr period (yes/no) and a categorical variable for the number of times they recalled eating rice over the same 24-hr period and tested for differences by migrant group using chi-squared tests.

### 4.2 Qualitative data analysis

We analyzed responses to open-ended questions regarding dress using open coding to identify concepts and group them into categories (Bryman, 2008). Similarly, we analyzed field notes pertaining to dress, food and socially maturing using grounded theory (Glaser and Strauss, 2019). We identified commonalities and outliers among observations and conversations, and then grouped them into themes.

### 4.3 Data integration

We triangulated questionnaire, biomarker and participant observation data. In Fig. 3, we present the data for biocultural markers as a joint display (Guetterman et al., 2015), with the quantitative results presented in bar charts according to migration scale and corresponding qualitative statements made by girls either living in Bangladesh or England in adjacent columns. Regarding Question 3, we predicted the median age of wearing the hijab (ever and daily), social correlates of puberty identified through participant observation and focus groups and quantified in the structured questionnaire, using the same Weibull models for the biological pubertal outcomes. We plotted median age of social correlates with the biological correlates in Fig. 4 and interpreted the observed patterns according to themes that emerged in the qualitative data.

## 5 Results

### 5.1 Does migration accelerate pubertal processes?

Table 1 reports the hazard ratios and 95% confidence intervals for earlier age at adrenarche, thelarche, and menarche across the four migration scale groups and the corresponding median ages derived from the Weibull models (medians are also depicted in Fig. 4). First-generation migrants reached adrenarche two years before all other girls (median age at adrenarche = 5.3 vs 7.1–7.4 years; p = 0.005 to 0.007). All three groups of girls living in the UK reached thelarche at younger ages than girls living in Sylhet (p for trend<0.001). Although not statistically significant, first-generation girls tended to reach thelarche earlier than second-generation girls (median age at thelarche = 9.2 vs 9.6 years; p = 0.055) and age at menarche was earliest in first-generation migrants (median age at menarche = 11.8 vs 12.1–12.6 years; p for trend = 0.64), although not statistically. First-generation girls also had the longest time-span between adrenarche and thelarche, also knowns as the juvenile tempo [Table 1: B = 3.5 (2.8–4.2), BB1 = 3.9 (−2.6-10.4), BB2 = 2.2 (1–3.4), WB = 1.6 (−0.9–4.1)].

**Table 1:** Timing and tempo of puberty by migration scale.

n	Bangladeshi	1st Generation		2nd Generation		White British		**p-trend**	**p-value for 1st vs 2nd**
	162	40		159		48			
**Pubertal Timing**	**Reference**	**HR (95%CI)**	**pairwise p**	**HR (95%CI)**	**pairwise p**	**HR (95%CI)**	**pairwise p**		
Adrenarche	**1**	**2.6 (1.3**–**5.0)**	**0.005**	**0.9 (0.6**–**1.3)**	**0.557**	**1.0 (0.6**–**1.7)**	0.868	0.707	0.007
Thelarche	**1**	**2.0 (1**–**4.1)**	**0.047**	**1.6 (1.1**–**2.4)**	**0.01**	**2.6 (1.5**–**4.4)**	<0.001	0.0003	0.553
Pubarche	**1**	**0.7 (0.4**–**1.5)**	**0.411**	**1.6 (1.0**–**2.4)**	**0.071**	**2.1 (1.1**–**3.7)**	0.018	0.009	0.032
Menarche	**1**	**1.8 (0.8**–**4)**	**0.183**	**1.4 (0.8**–**2.4)**	**0.245**	**0.9 (0.4**–**2.1)**	0.792	0.637	0.559
**Predicted Median Ages**	**Median Years**	**Median Years**		**Median Years**		**Median Years**			
Adrenarche	7.2	5.3		7.4		7.1			
Thelarche	10.7	9.2		9.6		8.7			
Pubarche	12.5	13.2		11.6		10.9			
Menarche	12.5	11.8		12.1		12.6			
**Tempo**	**Duration in years, 95% CI**	**Duration in years, 95% CI**		**Duration in years, 95% CI**		**Duration in years, 95% CI**			
Juvenile Tempo (Adrenarche to Thelarche)	**3.5 (2.8**–**4.2)**	**3.9 (-2.6**–**10.4)**		**2.2 (1**–**3.4)**		**1.6 (-0.9–4.1)**			
Pubertal Tempo (Thelarche to menarche)	**1.8 (1.1**–**2.5)**	**2.6 (-4.6**–**9.8)**		**2.5 (1.8**–**3.2)**		**3.9 (-3.9–7.8)**			

Higher BMI z-scores were significantly associated with earlier ages at thelarche, but not adrenarche or menarche (Table 2). When this association was examined within each migrant group, BMI only predicted earlier age of thelarche in Bangladeshi (p < 0.001) and white British girls (p = 0.01).

**Table 2:** Body mass index, overweight and obesity and the association (β* and P-value) between BMI z-scores and the timing of adrenarche, thelarche and menarche in Bangladeshi, First-Generation, Second-Generation and white British girls in the ABBY Project, derived from Weibull Models.

	N	Body Size		Pubertal Outcomes					
		BMI	Overweight/Obese	Adrenarche		Thelarche		Menarche	
		Mean (SD)	%	β	P	β	p	β	p
Overall	410	18.2 (3.9)	25%	0.08	0.16	0.29	<0.001	0.10	0.34
Bangladeshi	165	16.4 (3.4)	8.4%	0.09	0.37	0.48	0.001	0.38	0.08
First-Generation	39	19.6 (4.3)	35%	0.28	0.33	0.02	0.92	−0.23	0.50
Second-Generation	156	18.8 (3.6)	32%	0.07	0.49	0.06	0.59	−0.09	0.61
White British	50	19.9 (3.8)	48%	0.37	0.18	0.82	0.01	0.39	0.30

*a positive β represents an earlier age at pubertal outcome per unit increase in BMI z-score.

### 5.2 What biocultural markers are associated with migration?

**Fig. 2:**
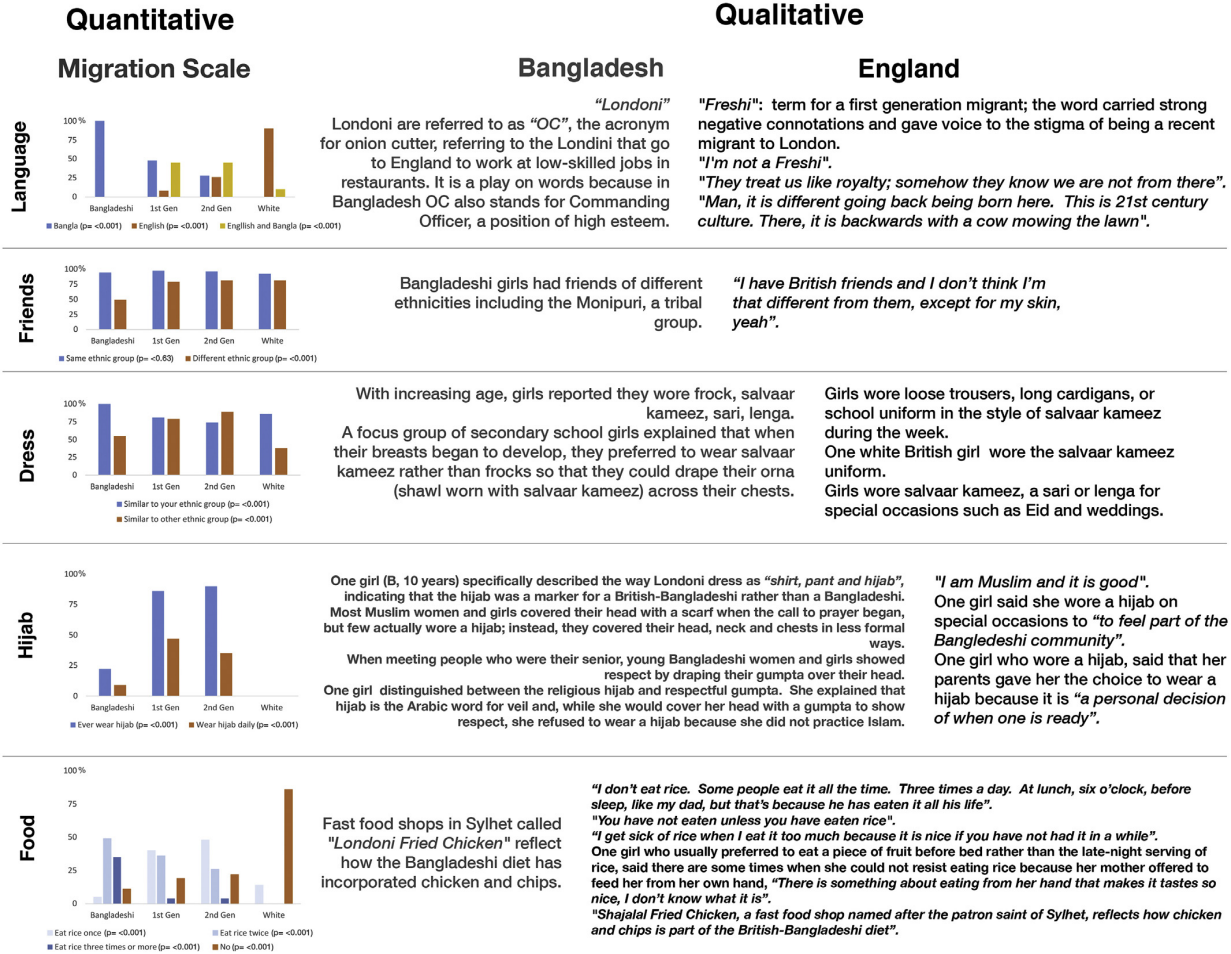
Joint quantitative and qualitative display of biocultural markers among Sylheti, First-generation, Second-generation and white British girls.

Fig. 2 presents the percentage of girls by migration scale reporting each biocultural marker derived from the questionnaire responses. In adjacent columns are corresponding qualitative data. We first describe the patterns of each marker by migration scale and then provide ethnographic detail for each.

#### 5.2.1 Language

Quantitative Results: The use of English language increased with migration scale, and the use of Bangla decreased (p < 0.001). Almost half (45%) of the first- and second-generation girls spoke Bangla and English equally when at home. Some white British girls reported speaking Bangla with their friends.

Qualitative Results: The words ‘Londoni’ and ‘Freshi’ and their connotations reflect nuances with regard to migration. In the Bangladeshi dialect, ‘Londoni’ referred to all Bangladeshis living in England, while the term for a first-generation migrant was ‘Freshi’. However, ‘Freshi’ carried strong negative connotations and gave voice to the stigma of being a recent migrant to London (Fig. 2). For example, at one secondary school, there was a group of ‘Freshi’ girls who had recently moved to East London from Bangladesh. We observed that they did not interact with other students even though the student body was 95% Bangladeshi. They spoke very little English and we observed that they were far behind in their coursework and the teachers were unable to give them extra attention during class time; this was observed to lead to further segregation as they sat together but away from other students in the class. Such segregation may be a source of psychosocial stress for first-generation migrants, although we did not inquire directly with the participants if this was the case.

Other ‘Freshis’ were observed to interact more with their peers and integrate into the British culture, especially if they had moved to the UK very early in life. One girl (BB1, aged 14 years) told her friends that she was born in Bangladesh, but moved to the UK when she was baby. Although she could hide being a ‘Freshi’, she asked her friends not to tell anyone that she was not born in the UK, indicating the stigma associated with being born in Bangladesh.

#### 5.2.2 Friends

Quantitative Results: The ethnic distribution of friends was similar across the migrant groups, with 92–97% of girls reporting that most of their friends were from the same ethnic group (p = 0.63). Compared with girls from all the groups living in the UK (first- and second-generation and white British girls), Bangladeshi girls reported having fewer friends from other ethnic groups (p < 0.001). However; they identified 49% of their friends as coming from other ethnic groups, suggesting additional ethnic differences beyond those we used to pre-define the groups (Bangladeshi vs British) (Fig. 2). For example, Bangladeshi girls made distinctions between being Muslim or Hindu as well as being indigenous.

Qualitative results: Even though the quantitative data suggest that the ethnic distribution of friends was similar across the migration scale, our qualitative data revealed that many girls did not understand the term ‘ethnicity’ and did not identify themselves or their friends as British or Bangladeshi.

#### 5.2.3 Clothing

Quantitative Results, Clothes: Style of dress became less similar to the same ethnic group in second-generation (74%) and first-generation (81%) girls compared to Sylheti girls (100%) (p < 0.001). Style of dress became more similar to other ethnic groups in second-generation (89%) and first-generation (79%) girls compared to Sylehti girls (55%) (Fig. 2; p < 0.001).

Hijab: Wearing hijab increased with migration scale (Fig. 2; p < 0.001). British-Bangladeshi girls reported wearing hijab (86–90%) more than Bangladeshi girls (21%).

Qualitative Results, Clothes: The proportion of British-Bangladeshi girls that reported wearing British clothes increased with migration scale according to the quantitatively assessed data from the structured questionnaire, but we observed that their choice and manner of wearing the clothes resembled Bangladeshi style and followed their interpretation of Islamic teachings. Only when British-Bangladeshi girls wore salvaar kameez (long tunic over trousers), a sari (long fabric pleated and wrapped around the body) or a lenga (sequined dress worn over fitted trousers) for special occasions such as *Eids* (religious holidays) and weddings would they think of themselves as ‘dressing like a Bangladeshi girl’ (Fig. 2). Regarding day to day clothes, some secondary schools had strict dress codes and provided a salvaar kameez in the school colors. In others, the dress code was more lenient and only required students to wear black. In these schools, the girls wore layers of clothes including loose fitting trousers and multiple tops (long-sleeved shirts under short-sleeved shirts), covered with long cardigans (Fig. 2). The clothes resembled a salvaar kameez as the trousers fit loosely and the long tops hung down to the mid-thigh.

**Fig. 3:**
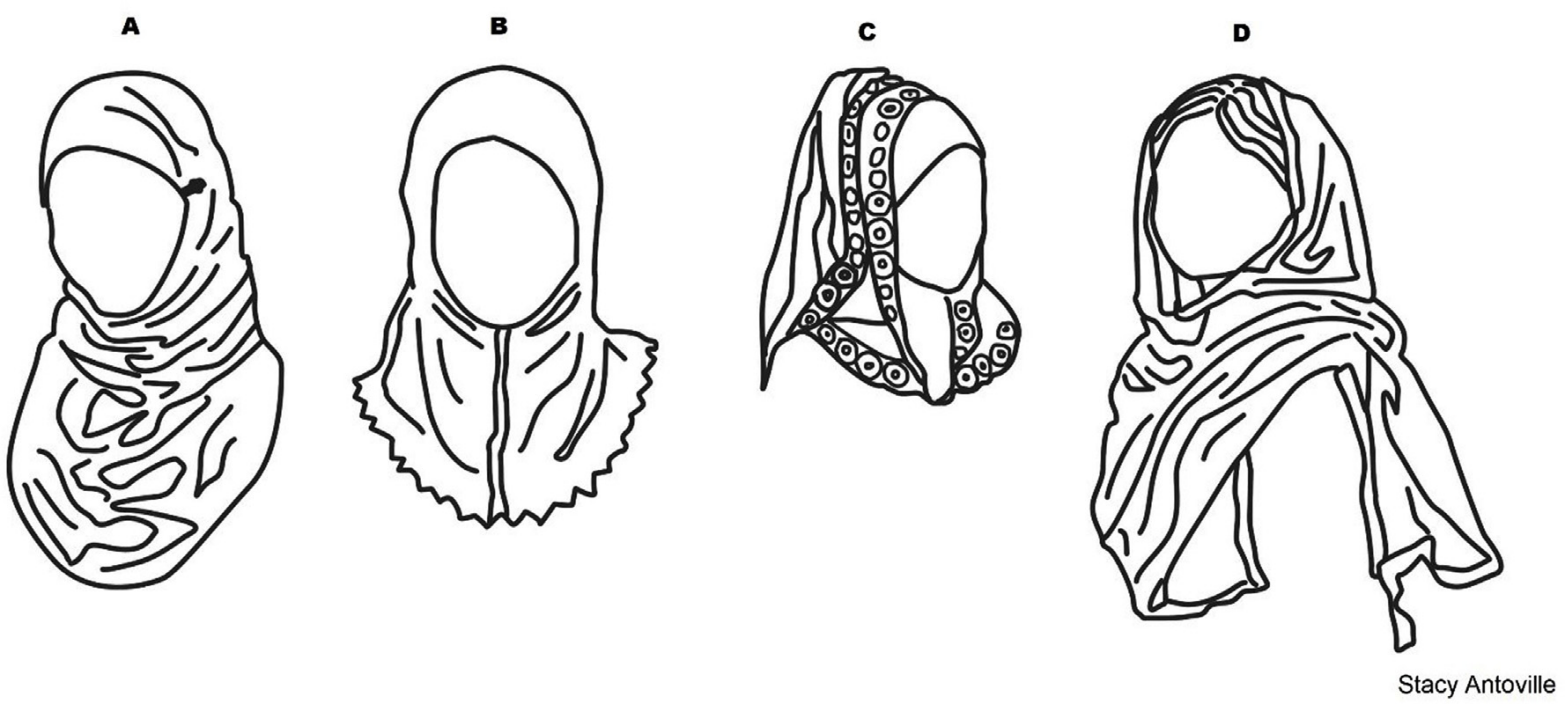
Hijab Styles. A) *hijab* scarf secured with pins B) pre-sewn *hijab* C) fashion *hijab* D) loose *gumpta* or *orna (illustration by Stacy Antoville)*.

Hijab: The proportion of girls wearing hijab did not increase with migration scale and our observations elaborate on these quantitative results. Second-generation girls explained that they wore hijab as a *Muslim* rather than as a *Bangladeshi*, a distinction that clearly mattered a lot to them. Despite religious prescriptions, like any piece of clothing, girls in either Bangladesh or the UK wore hijab in a number of ways depending on their personal style. The scarves varied from solid, plain colors like black, brown or beige to brightly colored with sparkly thread and designs. Most girls in the UK wore their scarves tightly wrapped around their head, face and neck, secured with pins (Fig. 3A). Some younger girls wore pre-sewn scarves that covered the head, neck and shoulders and were easily pulled-on and removed (Fig. 3B). Some teenaged girls arranged their hair so that a decorative scarf sat high on a bun that was covered by a tighter fitting scarf (Fig. 3C). Bangladeshi girls were less likely than first- and second-generation girls to wear hijab, as depicted by Fig. 3A-C. In place of hijab, most Bangladeshi girls temporarily covered their head a loosely draping cloth—either their *orna* (the shawl worn with a salvaar kameez), or a scarf worn with the school uniform—around their shoulders and neck and pulling it up from behind (Fig. 3D). For most of the day, they did not cover their head and left their scarf hanging across the shoulders. Given the variation in styles of hijab, comparing the proportion of girls that wear hijab across the migration scale misses the girls’ flexibility and creativity in how they wear it and what it means in terms of ethnic identity.

#### 5.2.4 BMI and food

Quantitative Results: The proportion of overweight and obesity increased with migration scale (Table 2: B = 8.4%, BB1 = 35%, BB2 = 32%, WB = 48%; p < 0.001). Eating rice is a central part of a Bangladeshi meal and the word ‘rice’ encompasses the other food preparations such as meat and/or vegetable curries eaten with rice. The proportion of girls that did not eat rice within 24-h of recall increased with migration scale (p < 0.001). While first- and second-generation British-Bangladeshi girls ate rice fewer times per day than Bangladeshi girls, a vast majority of British-Bangladeshi girls (80%) ate rice at least once a day (Fig. 3; p < 0.001).

Qualitative Results: Contrary to the high percentage of girls who reported eating rice in the questionnaire, we observed British-Bangladeshi girls flippantly saying: “I don't eat rice” or “I never eat rice”, when talking to each other. When asked to explain this apparent discrepancy between their 24-h food recall and their conversations with friends, some girls contradicted themselves in their explanation, “I don't eat it, but I had it yesterday”, or: “I haven't eaten it in a month, but I had it yesterday”. For these girls, the phrase, “I don't eat rice” was not literal, but rather reflected that she ate rice less frequently than those in Bangladesh, and was a way to reject being Bangladeshi. However, some girls acknowledged that, at times, they enjoyed eating rice, such as after not having it in a while or when eating from a mother's hand (a mother feeding her children from her own hand is a common practice among young children in Bangladesh (Ashraful Aziz and Maloney, 1985) (Fig. 2).

Instead of rice, girls said that they preferred eating foods that could be considered more ‘British’. One girl (BB2, aged 14 years) requested that her mother prepare “pasta, lasagna, prawns, salad, garlic bread or breaded fish” instead of rice. Many participants in the UK reported eating chicken and chips. The reported preference for this fast, prepared food was as much about taste and preference as it was about a way to spend time with friends without parents worrying. In fact, many chicken and chip shops were owned by Sylhetis and specialized in serving halal chicken. For example, one of these shops was called Shahjalal Chicken, named after the patron saint of Sylhet. Similarly in in Bangladesh, the diet included chicken and chips, and in Sylhet, there was a chain named Londoni Fried Chicken. While migrant groups reportedly ate less rice than those in Sylhet, they *added* British foods to their diet rather than *replacing* rice altogether, resulting in similar dietary patterns between Bangladeshis and migrant groups. However, the consumption of rice *and* British foods may explain the higher BMI observed among first- and second-generation girls when compared to Bangladeshis.

Summary of Integrated Results: Overall, the discrepancies between the quantitative and qualitative markers are captured by what British-Bangladeshi girls often said: “I am proud of my religion but not my culture”. Thus, they denied and rejected behaviors that they attributed to ‘Bangladeshi culture’, such as wearing salvaar kameez and eating rice, but they embraced behaviors that they attributed to ‘religion’, such as wearing hijab and eating halal food.

## 6 Biocultural markers of puberty

To address the third question: "What biocultural markers are associated with puberty?", we compared style of dress and wearing hijab with pubertal development across the four migration groups.

### 6.1 Clothes

In Bangladesh, the type of clothes girls wore tracked with pubertal development, with changes occurring alongside major pubertal milestones. During a secondary school focus group in Sylhet, girls explained that the style of clothes that Bangladeshi girls and women wore changed over the life course: young girls wore skirts, frocks or pant at age five, older girls wore salvaar kameez around age 10, and women wore saris once married or working in professional jobs. In other words, the transition from wearing a frock to a salvaar kameez links with the onset of puberty. A focus group of secondary school girls in Bangladesh (ages 10–13 years) explained that, when their breasts began to develop, they preferred to wear salvaar kameez rather than frocks so that they could drape their *orna* (shawl worn with salvaar kameez) across their chests. We observed this social marker of puberty while conducting fieldwork in Sylhet, but because we conducted the fieldwork in East London first, we were unable to investigate whether styles of clothes changed with the onset of breast development among Bangladeshi migrant groups in the UK.

### 6.2 Hijab

**Fig. 4:**
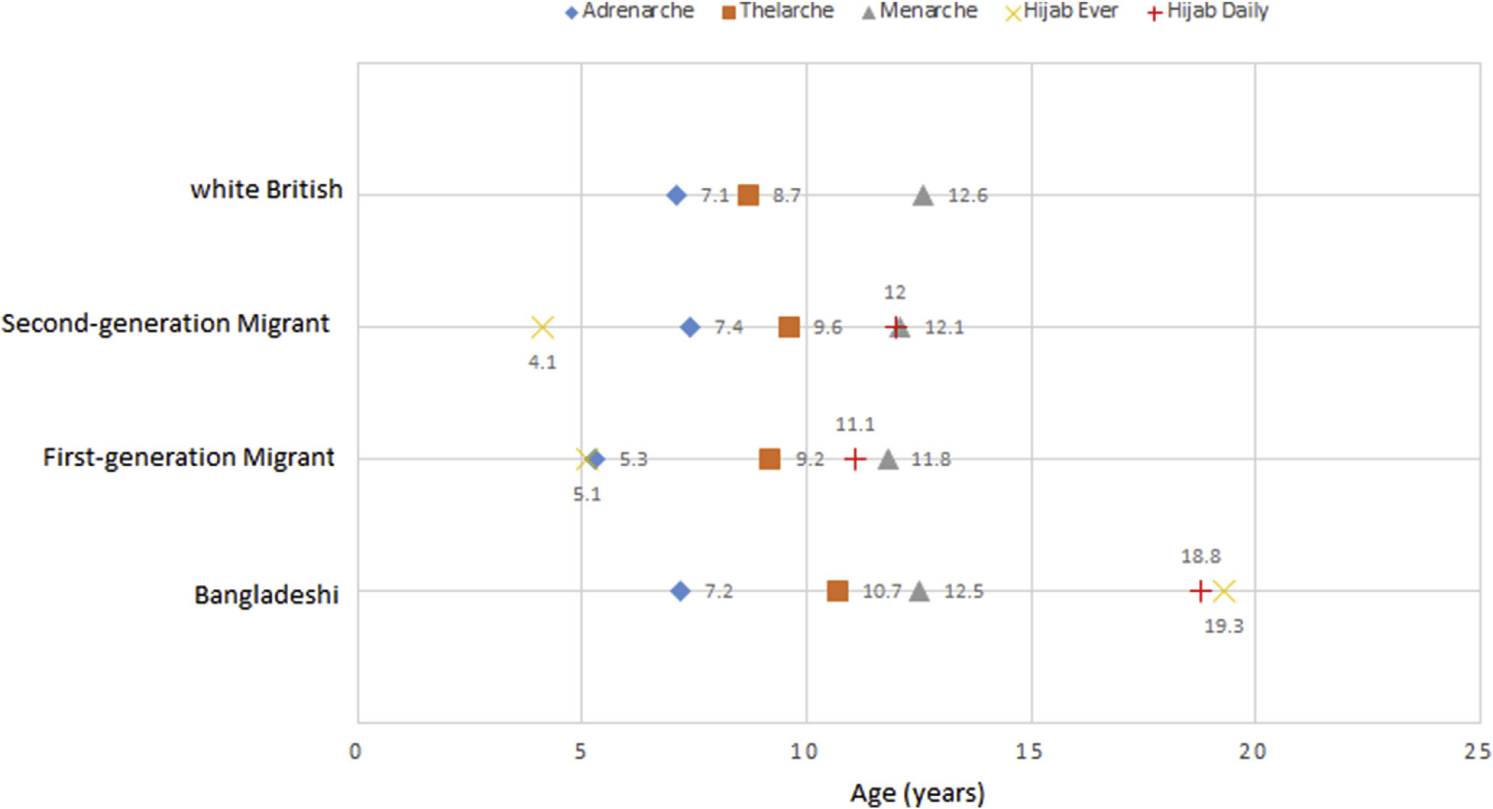
Median ages of biological and social correlates of puberty in bangladeshi, first-generation, second-generation, white british girls in the ABBY project.

Among Bangladeshi girls, the median age of wearing hijab ever or daily occurred years after the onset of all pubertal outcomes (Fig. 4). In contrast, for first-generation girls, ever wearing hijab (age 5.1) coincided with the age at adrenarche (age 5.3), whereas among the second-generation girls, ever-wearing hijab (age 4.1) occurred 3.3 years before adrenarche (age 7.4). The ages at which first- and second-generation girls started to wear hijab every day, 11.1 and 12.0 years, coincided with their ages at menarche, 11.8 and 12.1, respectively (Fig. 4).

British-Bangladeshi girls explained the distinction between *ever* wearing and *always* wearing hijab as “practicing” in the process of becoming “dedicated to the scarf”. Girls who did not wear hijab every day were practicing, while girls that wore hijab every day were dedicated. The beginning of the practicing stage coincides with adrenarche, when the capacity for social learning emerges. One girl (BB2 aged 14 years), who wore her hijab every day, explained:

“Being dedicated to the hijab means being in control of yourself, getting yourself sorted, behaving like a good Muslim … If a girl is hanging out with a group of boys and smoking than she shouldn't be wearing the scarf.”

She further explained that “it is inside what counts”, and some girls wear hijab for fashion, but this is not “being dedicated”. A younger girl spoke of playing dress-up and putting on hijab to be like a bride. Other girls spoke of the practicing stage as “getting used to it”. Some girls chose to become “dedicated” to the hijab when they transitioned to secondary school, or in their final years at secondary school, while others were still “practicing”. One girl (BB2, 15 years old), confidently explained that the reason she does not wear hijab was that she simply was “not ready”. Rather than wear hijab and contradict what it represented, she chose not to wear it. However, for her and those still practicing, there was an underlying expectation in the UK to become "dedicated" at some point. While there were variations in the meanings and timing, learning to wear hijab was part and process of socially maturing as a British-Bangladeshi girl. This is in sharp contrast to the context in Sylhet, where some adult women wore hijab and others did not, and where the median age for wearing hijab (ever or daily) was starkly older than in the UK (18–19 years vs. 4–12 years, p < 0.001; Fig. 4).

## 7 Discussion

The results of this biocultural study of puberty among Bangladeshi migrants to the UK collectively suggest that effects of migration are embodied and alter the timing of pubertal development for first-generation migrant girls. Stress and growth are two established factors affecting the timing of puberty. (Greenspan and Deardorff, 2014) Here we demonstrate that the stress effects appear to be strongest during adrenarche, when adrenal hormones are first produced by a mature HPA axis. The growth effects, measured as BMI, are strongest on the timing of thelarche, but only in the non-migrants. Embodied culture shock among first-generation migrants accelerated the biological timing of puberty, and lengthened the social maturation period between adrenarche and menarche, when healthy behaviors can be practiced and eventually adopted.

The answer to the first question, ‘Does migration accelerate pubertal processes?‘, is true for first-generation migrant girls. The phrase “I am not a Freshi” offers one explanation. First-generation girls (known as ‘Freshis’ in the UK) had an accelerated timing of adrenarche, thelarche and menarche in relation to Bangladeshi and second-generation girls. First-generation girls were distinguished from the others in that they experienced a marked change in growth in height, weight, and BMI and psychosocial factors early in life. We have previously shown that first-generation girls underwent rapid catch up growth (Houghton et al., 2014), and our qualitative data indicate that the negative connotations around being a ‘Freshi’ may be a source of psychosocial stress. The resulting psychosocial stress that accompanied migration may have led to an earlier activation of the hypothalamus-pituitary-adrenal or stress axis and, in turn, an earlier adrenarche, since stress has been shown to increase androgen levels (van Zuiden et al., 2017). Elevated pre-pubertal androgens accelerate the timing of thelarche (Remer et al., 2010), which could also explain the earlier age of thelarche in first-generation compared to second-generation girls. Our findings are consistent with the “biological sensitivity to context” theory which has been postulated to explain earlier pubertal maturation among girls who experience stress within their households (Belsky, 2000; Boyce and Ellis, 2005). In the case of migration, having elevated stress due to stigma of being a recent migrant may lead to higher susceptibility to the biological environment in the UK. Mixing quantitative and qualitative data, we can now contribute an integrated hypothesis to both psychological and biological studies investigating the drivers of pubertal timing. Specifically, we hypothesize that stress increases androgen concentrations, which are converted in adipose tissue to estrogens leading to earlier breast development. We propose this hypothesis as one mechanism of embodiment. Alternate explanations for elevated androgens observed in first generation girls include prenatal factors (Cohn and Cirillo, 2019), consumption of animal protein (Shi et al., 2009) or greater adrenal sensitivity to stress (Kamin and Kertes, 2017).

The identification of biocultural markers of migration is complex, as illustrated by the words of second generation girls words, “I am proud of my religion but not my culture”. The rejection of Bangladeshi culture was evident in the girls' denial of participating in Bangladeshi behaviors such as eating rice. Not only did British-Bangladeshi girls reject behaviors associated with Bangladesh, but they also separated themselves from people who had recently migrated from Bangladesh by mockingly referring to them as ‘Freshis’. Still, they were proud to be Muslim. The clearest marker of their devotion to Islam was their choice to wear hijab. Jacobson (2006) similarly observed that Pakistani Youth in London identified with their religion more than their ethnicity or nationality, and concluded that some markers of identity are more fluid than others (Jacobson, 2006). Dress among British-Bangladeshi girls is what Jacobson refers to as a fluid marker of identity, in that scarves, trousers and tops bought in typical British stores satisfied prescribed Bangladeshi norms but were worn in ways that appealed to a new urban, young and Muslim aesthetic. By rejecting and accepting certain behaviors associated with either their country of origin or host country, British-Bangladeshi girls negotiated who they were and who they were becoming and, in turn, created identities beyond the scope of traditional acculturation markers in surveys. We conclude that they did this through operationalizing “habitus”; defined as the values and dispositions gained from cultural histories that can be applied across contexts (Webb et al., 2002). Bourdieu maintains that these values are both durable and transposable, and migrant girls were equipped with the tools to respond appropriately to new situations. But their responses were largely regulated by where (and who) they have been in a culture (Webb et al., 2002; Bourdieu and Nice, 1977). Appadurai qualifies Bourdieu's definition of habitus by suggesting that globalization affects habitus by increasing the rate of improvisation (Appadurai, 1997). In the context of migration, the movement through different cultural fields requires constant improvisation, which explains how biocultural markers may not fully capture behaviors among migrant groups especially during a significant period of identity development, such as adolescence.

We answered our third question, “What biocultural markers are associated with puberty?” through observing the process of “practicing to becoming dedicated to the scarf.” For British-Bangladeshis, among whom almost all wore hijab, the transition from practicing to becoming dedicated encapsulated the biocultural process of maturing from a girl to a woman. Among British-Bangladeshi girls, the stage of “practicing” aligned with the emergence of adrenarche. Becoming ‘dedicated to the hijab’ was more a gradual process and aligned with menarche for some but not all girls.

The correlation of practicing hijab with the onset of adrenarche makes sense in terms of biology and culture. Biologically, adrenarche and the corresponding brain changes equips humans with the skills necessary for future social interactions (Campbell, 2006b) and begins an essential period for social learning. Culturally, adrenarche is also the beginning of middle childhood. Lancy and Grove (2011) compared markers of middle childhood across cultures and observed a universal shift in behavior that in turn is noticed by adults, albeit less ceremonially than the arrival of puberty (Lancy and Grove, 2011). During middle childhood, the social interactions of a child extend from the family to the neighborhood context and this extension increases opportunity to learn cultural rules and behaviors, such as caring for siblings, carrying wood, participating in food production, and going to school (Weisner et al., 1977; Blurton-Jones et al., 1997). “Practising” hijab by many of the adenarcheal girls in ABBY is a manifestation of this advance in understanding.

### 7.1 Public health implications

The majority of discourse about puberty revolves around the worry of what earlier maturation means socially and biologically. Earlier maturation in girls has been associated with lower self-esteem, a less favorable body image, greater rates of eating problems, depression, suicide attempts, greater association with deviant peers, norm-breaking behaviors and earlier onset of sexual intercourse and greater risk for chronic diseases (Biro et al., 2010). As Worthman (1999:152) writes, “Clinicians and the public alike are continually amazed at the psychological and behavioral precocities of contemporary youth and remain conditioned by schedules of childhood and youth that are dissonant with current patterns of maturation” (Worthman, 1999). Rather than highlight early maturation in a negative way, it is helpful to disassociate it from age, as social theorists of childhood suggest (James et al., 1998) and to view juvenility as a malleable stage during which health promotion could have lasting impact.

There is cross-cultural recognition of increased childhood social abilities after adrenarche (Lancy and Grove, 2011), but public taboos and concerns about the juvenile period are more or less non-existent compared to the concerns about premature puberty which circulate in the academic and lay arenas. Campbell and colleagues refer to adrenarche as the beginning of “prepubescent adolescence” dislodging adrenarche from the social stigma associated with puberty (Campbell, 2006a). If adrenarche marks the increased capacity of the sense of self, and adolescence marks the making of that self, then the period of development between them (when children are learning and practicing social behaviors) is a critical time during which healthy behaviours are established and track into adolescence and adulthood. As evidenced by the discordance between the denial of eating rice but actually eating it and practicing hijab and becoming dedicated to it, girls learned and experimented with behaviors during the juvenile and pubertal windows. Intervening on common drivers of early puberty during the juvenile period would not only alter pubertal timing but also have lasting implications for adult health and disease.

### 7.2 Limitations

Our findings should be considered within the limitations of the study. We designed the study to optimize the representation of the different groups, but sample size was not equally distributed. The small sample of first-generation and white British girls may have resulted in false positive findings (in the case of age at adrenarche) or undetected differences (in the case of menarche). The convenience sampling approach may have led to selection bias in that our recruitment methods may have appealed to those comfortable stating their migration status. Because of integrated mixed-method study design (Zhang and Creswell, 2013) and the simultaneous collection of quantitative and qualitative data, we were not able to investigate all qualitative themes quantitatively and vice versa. For example, we were not able to track type of clothes with age in British-Bangladeshi girls, because this theme emerged in Bangladesh after we conducted fieldwork in the UK. Lastly, biocultural markers change over time and potentially, at different rates within an individual's lifespan (Fox et al., 2017) and so the snapshot presented here from girls and adolescents during a time of identity development should not be interpreted as fixed or stagnant. Furthermore, whether the differences in biological and social pubertal timing represent latent, cumulative, or pathway effects in relation to long term health cannot be determined using our cross-sectional study design, and longitudinal follow up later in the life course is warranted. For example, whether the extended juvenile period in first-generation girls and the corresponding extended period of social learning reflect common underlying causes or are merely coincidentally associated must be determined through longitudinal studies. However, an important strength of our study is the inclusion of Bangladeshi girls, as most migrant studies lack a comparison group from the country of origin.

## 8 Conclusion

Our results are consistent with the hypothesis that the effects of migrating, through stress and growth, are embodied by girls, and in turn accelerate and lengthen the juvenile period. An earlier and longer juvenile period extends the period of learning and increases the period of transition, when the adoption of positive behaviors can modify childhood trajectories towards health and wellbeing (Viner et al., 2012). Our integrated quantitative and qualitative findings support complementary theories that both stress and growth affect the timing of puberty. Lifestyle factors, such as language, friendship, dress and food among migrant groups did not merge to match British ones in a unidirectional way. Migration did not result in becoming more ‘British’, but rather girls improvised and negotiated identity options that resulted in fluid identities unique to British-Bangladeshi girls, that at times contradicted their reported behaviors. Identifying with British-Bangladeshi girls was particularly difficult for first-generation girls within the British-Bangladeshi community, and may be a source of psychosocial stress, which may explain their accelerated pubertal development. Growth, measured as body mass index, was an important predictor of puberty for Bangladeshi and British girls. These results, derived from mixed-methods point to testable biocultural hypotheses for future studies interested in how culture gets beneath the skin.

## Credit author statement

Lauren Houghton: Conceptualization, Data curation, Methodology, Formal analysis, Writing- Original draft preparation, Rebecca Troisi: Supervision, Writing- Reviewing and Editing, Hormuzd Katki: Formal analysis, Supervision, Writing- Reviewing and Editing, Marni Sommer: Methodology, Writing- Reviewing and Editing, Osul Choudhury: Data curation, Writing- Reviewing and Editing Mark Booth: Supervision, Methodology, Writing- Reviewing and Editing, Kate Hampshire: Methodology, Formal analysis, Supervision, Writing- Reviewing and Editing.

## References

[bib33] Ashraful Aziz KM, Maloney Clarence (1985). Life Stages Gender and Fertility in Bangladesh.

[bib3] Appadurai A. (1997). Modernity at Large : Cultural Dimensions of Globalization.

[bib4] Belsky J. (2000). Conditional and alternative reproductive strategies: individual differences in susceptibility to rearing experiences. Genetic Influences on Human Fertility and Sexuality.

[bib5] Belsky J., Ruttle P.L., Boyce W.T., Armstrong J.M., Essex M.J. (2015). Early adversity, elevated stress physiology, accelerated sexual maturation, and poor health in females. Dev. Psychol..

[bib6] Bhui K., Stansfeld S., Head J. (2005). Cultural identity, acculturation, and mental health among adolescents in east London's multiethnic community. J. Epidemiol. Community Health.

[bib7] Biro F.M., Lucky A.W., Simbartl L.A. (2003). Pubertal maturation in girls and the relationship to anthropometric changes: pathways through puberty. J. Pediatr..

[bib8] Biro F.M., Galvez M.P., Greenspan L.C. (2010). Pubertal assessment method and baseline characteristics in a mixed longitudinal study of girls. Pediatrics.

[bib9] Biro F.M., Greenspan L.C., Galvez M.P. (2013). Onset of breast development in a longitudinal cohort. Pediatrics.

[bib10] Blakemore S.-J., Mills K.L. (2014). Is adolescence a sensitive period for sociocultural processing?. Annu. Rev. Psychol..

[bib11] Blurton-Jones N.G., Hawkes K., O’Connell J.F., Segal N.L., Weisfeld G., Weisfeld C.C. (1997). Why do Hadza children forage?. Uniting Psychology and Biology : Integrative Perspectives on Human Development.

[bib12] Bodicoat D.H., Schoemaker M.J., Jones M.E. (2014). Timing of pubertal stages and breast cancer risk: the Breakthrough Generations Study. Breast Cancer Res..

[bib13] Bourdieu P., Nice R. (1977). Outline of a Theory of Practice.

[bib14] Boyce W.T., Ellis B.J. (2005). Biological sensitivity to context: I. An evolutionary-developmental theory of the origins and functions of stress reactivity. Dev. Psychopathol..

[bib15] Bryman A. (2008). Social Research Methods.

[bib16] Campbell B. (2006). Adrenarche and the evolution of human life history. Am. J. Hum. Biol..

[bib17] Campbell B. (2006). Adrenarche and the evolution of human life history. Am. J. Hum. Biol..

[bib18] Cohn B.A., Cirillo P.M. (June 2019). In utero and postnatal programing of dehydroepiandrosterone sulfate (DHEAS) in young adult women. Reprod. Toxicol..

[bib19] Cole T.J., Green P.J. (1992). Smoothing reference centile curves: the lms method and penalized likelihood. Stat. Med..

[bib20] Cole T.J., Freeman J.V., Preece M.A. (1998). British 1990 growth reference centiles for weight, height, body mass index and head circumference fitted by maximum penalized likelihood. Stat. Med..

[bib21] Deardorff J., Ekwaru J.P., Kushi L.H. (2011). Father absence, body mass index, and pubertal timing in girls: differential effects by family income and ethnicity. J. Adolesc. Health.

[bib22] Fox M., Thayer Z., Wadhwa P.D. (2017). Acculturation and health: the moderating role of socio-cultural context. Am. Anthropol..

[bib23] Gilbert P.A., Khokhar S. (2008). Changing dietary habits of ethnic groups in Europe and implications for health. Nutr. Rev..

[bib24] Glaser BG, Strauss AL. The Discovery of Grounded Theory : Strategies for Qualitative Research. https://books.google.com/books?id=C5QiwAEACAAJ&dq=editions:5Hs7DU0I0egC&hl=en&sa=X&ved=0ahUKEwjXxPC6svjhAhUDneAKHRatCwsQ6AEIUDAG. Accessed April 30, 2019.

[bib25] Greenspan L, Deardorff J (2014). The New Puberty : How to Navigate Early Development in Today’s Girls.

[bib26] Guetterman T.C., Fetters M.D., Creswell J.W. (2015). Integrating quantitative and qualitative results in health science mixed methods research through joint displays. Ann. Fam. Med..

[bib56] Havelock JC, Auchus RJ, Rainey WE (2004). The rise in adrenal androgen biosynthesis: Adrenarche.. Semin. Reprod. Med..

[bib27] Herman-Giddens M.E., Slora E.J., Wasserman R.C. (1997). Secondary sexual characteristics and menses in young girls seen in office practice: a study from the Pediatric Research in Office Settings network. Pediatrics.

[bib28] Herman-Giddens M.E., Kaplowitz P.B., Wasserman R. (2004). Navigating the recent articles on girls’ puberty in Pediatrics: what do we know and where do we go from here?. Pediatrics.

[bib2] Houghton LC, Cooper GD, Booth M (2014). Childhood environment influences adrenarcheal timing among first-generation Bangladeshi migrant girls to the UK. PLoS One.

[bib1] Houghton LC., Cooper GD., Bentley GR. (2014). A migrant study of pubertal timing and tempo in British-Bangladeshi girls at varying risk for breast cancer. Breast Cancer Res.

[bib29] Jacobson J. (2006). Islam in Transition : Religion and Identity among British Pakistani Youth.

[bib30] James A., Jenks C., Prout A. (1998). Theorizing Childhood.

[bib31] Kamin H.S., Kertes D.A. (2017). Cortisol and DHEA in development and psychopathology. Horm. Behav..

[bib32] Kaplowitz P. (2006). Pubertal development in girls: secular trends. Curr. Opin. Obstet. Gynecol..

[bib34] Krieger N. (2016). Living and dying at the crossroads: racism, embodiment, and why theory is essential for a public health of consequence. Am. J. Publ. Health.

[bib35] Krieger N., Davey Smith G. (2004). "Bodies count," and body counts: social epidemiology and embodying inequality. Epidemiol. Rev..

[bib36] Lancy D.F., Grove M.A. (2011). Getting noticed. Middle childhood in cross-cultural perspective. Hum. Nat..

[bib37] Lee Y., Styne D. (2013). Influences on the onset and tempo of puberty in human beings and implications for adolescent psychological development. Horm. Behav..

[bib38] Lofink H.E. (2012). ‘The worst of the Bangladeshi and the worst of the British’: exploring eating patterns and practices among British Bangladeshi adolescents in East London. Ethn. Health.

[bib39] Lofland J., Lofland J. (2006). Analyzing Social Settings : A Guide to Qualitative Observation and Analysis.

[bib40] Marshall W.A., Tanner J.M. (1969). Variations in pattern of pubertal changes in girls. Arch. Dis. Child..

[bib41] McDonald J.A., Eng S.M., Dina O.O., Schooling C.M., Terry M.B. (2016). Infection and pubertal timing: a systematic review. J. Dev. Orig. Health Dis..

[bib42] Perkins J.M., Subramanian S.V., Davey Smith G., Özaltin E. (2016). Adult height, nutrition, and population health. Nutr. Rev..

[bib43] Petersen A.C., Crockett L., Richards M., Boxer A. (1988). A self-report measure of pubertal status: reliability, validity, and initial norms. J. Youth Adolesc..

[bib44] Reiter E.O., Fuldauer V.G., Root A.W. (1977). Secretion of the adrenal androgen, dehydroepiandrosterone sulfate, during normal infancy, childhood, and adolescence, in sick infants, and in children with endocrinologic abnormalities. J. Pediatr..

[bib45] Remer T., Shi L., Buyken A.E., Maser-Gluth C., Hartmann M.F., Wudy S.A. (2010). Prepubertal adrenarchal androgens and animal protein intake independently and differentially influence pubertal timing. J. Clin. Endocrinol. Metab..

[bib46] Royston P. (2001). Flexible parametric alternatives to the Cox model, and more. Stata J. Promot. Commun. Stat Stata.

[bib47] Shi L., Wudy S.A., Buyken A.E., Hartmann M.F., Remer T. (2009). Body fat and animal protein intakes are associated with adrenal androgen secretion in children. Am. J. Clin. Nutr..

[bib48] van Zuiden M., Haverkort S.Q., Tan Z., Daams J., Lok A., Olff M. (2017). DHEA and DHEA-S levels in posttraumatic stress disorder: a meta-analytic review. Psychoneuroendocrinology.

[bib49] Viner R.M., Ozer E.M., Denny S. (2012). Adolescence and the social determinants of health. Lancet (London, England).

[bib50] Webb J., Schirato T., Danaher G. (2002). Understanding Bourdieu.

[bib51] Weisner T.S., Gallimore R., Bacon M.K. (1977). My brother's keeper: child and sibling caretaking [and comments and reply]. Curr. Anthropol..

[bib54] Worthman C., Trevathan W., Smith E.O., McKenna J.J., James J. (1999). Evolutionary perspectives on the onset of puberty. Evolutionary Medicine.

[bib52] Worthman C.M., Costello E.J. (2009). Tracking biocultural pathways in population health: the value of biomarkers. Ann. Hum. Biol..

[bib53] Worthman C.M., Trang K. (2018). Dynamics of body time, social time and life history at adolescence. Nature.

[bib55] Zhang W., Creswell J. (2013). The use of “mixing” procedure of mixed methods in health services research. Med. Care.

